# Tricyclic Derivative of Acyclovir and Its Esters in Relation to the Esters of Acyclovir Enzymatic Stability: Enzymatic Stability Study

**DOI:** 10.3390/molecules25092156

**Published:** 2020-05-05

**Authors:** Izabela Muszalska-Kolos, Monika A. Lesniewska-Kowiel, Szymon Plewa, Agnieszka Klupczyńska

**Affiliations:** 1 Chair and Department of Pharmaceutical Chemistry, Poznan University of Medical Sciences, 6 Grunwaldzka Str., 60-780 Poznań, Poland; lesniewska_monika@wp.pl; 2 Chair and Department of Inorganic and Analytical Chemistry, Poznan University of Medical Sciences, 6 Grunwaldzka Str., 60-780 Poznań, Poland; splewa@ump.edu.pl (S.P.); aklupczynska@ump.edu.pl (A.K.)

**Keywords:** tricyclic analogues of acyclovir, enzymatic hydrolysis, HPLC, HPLC-MS/MS

## Abstract

The 3,9-dihydro-3-[(2-hydroxyethoxy)methyl]-6-(4-methoxyphenyl)-9-oxo-5*H*-imidazo[1,2-*a*]–purine (6-(4-MeOPh)-TACV) was selected to assess the enzymatic stability of the tricyclic acyclovir derivatives from the imidazo[1,2-*a*]-purine group. The parent compound and its esters (acetyl, isobutyryl, pivaloyl, nicotinic, ethoxycarbonyl) were subjected to kinetic studies and compared with the stability of analogous acyclovir (ACV) esters. The enzymatic hydrolysis was observed in vitro in a medium of 80% human plasma in the absence and presence of porcine liver esterase (PLE). The tests were carried out at 37 °C. To determine the kinetic parameters (k_obs._, t_0.5_) of the observed reaction, the validated HPLC-UV method in the reversed phase was used. The HPLC-MS/MS method was used to identify the degradation products under the tested conditions. In summary, it was found that 6-(4-MeOPh)-TACV esters are more susceptible to esterase metabolism than ACV esters. It was confirmed by HPLC-MS/MS that in the plasma, the main product of their hydrolysis is 6-(4-MeOPh)-TACV and not ACV, which confirms that their antiviral activity observed in vitro does not result from ring degradation.

## 1. Introduction

Since the 1960s, there has been a steady increase in interest in the preparation and use of prodrugs. The purpose of the prodrug design is to optimize the properties of the compounds exerting the desired pharmacological action that cause problems with the further development of the drug. This applies mainly to the solubility or lipophilicity of the active substance. By definition, a prodrug is an inactive form of the drug. Therefore, the key to achieving a pharmacological effect is its activation in the body. The vast majority of prodrugs undergo enzymatic activation, most often with the participation of hydrolases or cytochrome P450 [1,2] enzymes. In the case of systemically acting systemic drugs, the resulting prodrug is usually intended to be a substrate for hydrolases that commonly occur and accept a variety of substances, such as peptidases, phosphatases, and especially esterases.

Many prodrugs have an ester group in the structure. These include, among others, penicillin (bacampicillin, pivampicillin), cephalosporin (cefethoxime axetil, cefetamet pivoxil), macrolide (cyclic erythromycin carbonate), vitamin (retinol acetate, α-tocopherol acetate), β-adrenolytic (timolol benzoate), and β-adrenergic (ibuterol, bambuterol) drugs [3]. Removal of the ester bond occurs most often with the participation of esterases, commonly occurring in the body, such as carboxyl esterases, acetylcholinesterases (AChE), butyrylcholinesterase (BChE), paraoxonase**, and arylesterase. It is also possible to break down the ester bond due to the oxidation of the cytochrome P450-catalyzed enzyme [2]. Prodrugs activated by carboxyl esterases include capecitabine [4], paclitaxel 2-ethyl carbonate [5], and propranolol ester prodrugs [6]. AChE also participates in the activation of prodrugs [2,7], such as esters of propranolol [6], acyclovir [8], and dipivefrin hydrochloride [9]. The butyrylcholinesterase also has high catalytic activity, but it is selective for butyrylcholine and propionylcholine. It is characterized by a substrate specificity that is wider than AChE. It is produced in the liver and reaches its highest concentration in the plasma. BChE hydrolyses many esters and, therefore, it is also involved in the detoxification processes of xenobiotics and plays an important role in the metabolism of, among others, local anesthetics [10], succinylcholine [11], aspirin [12], or heroin [13]. In addition, it participates in the activation of prodrugs, such as propranolol esters [6], bambuterol [14], methylprednisolone acetate [15], and isosorbide diaspirate [16]. Despite the albumin, the activity of esterases also present carboxypeptidase A [17], aldehyde dehydrogenase [18], carbonate anhydrases B and C [19], trypsin [20], and lipase [21], which should be considered when designing ester prodrugs.

For the treatment of herpes simplex type 1 and 2 and herpes zoster, the most commonly used drug is acyclovir (ACV) and the prodrugs valaciclovir (VACV) and famciclovir (FCV), which represent a breakthrough in antiviral therapy. However, increasing drug resistance has resulted in a decrease in their effectiveness to below 0.5%. In patients with reduced immunity, drug resistance is more common; therefore, it is desirable to look for alternative drugs with acceptable safety profiles. Similar conclusions apply to the treatment of cytomegalovirus and in this case, ganciclovir (GCV) and valganciclovir (VGCV) are used [22].

**Figure 1:**
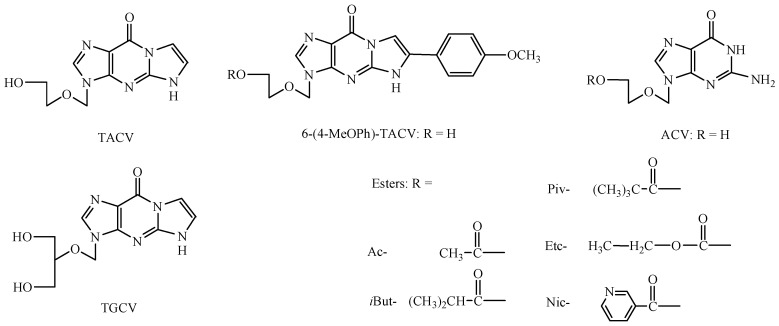
The chemical structure of 3,9-dihydro-3-[(2-hydroxyethoxy)-methyl]-9-oxo-5*H*-imidazo[1,2-dihydroxy-1,2-*a*]purine derivatives as a modification of acyclovir (TACV), ganciclovir (TGCV), and the tested esters of 3,9-dihydro-3-[(2-hydroxyethoxy)methyl]-6-(4-methoxyphenyl)-9-oxo-5*H*-imidazo[1,2-*a*]–purine (6-(4-MeOPh)-TACV) and acyclovir (ACV).

Modifications within the acyclovir (ACV) base part have resulted in further monocyclic derivatives (derivatives of isocytosine, triazole, imidazole), bicyclic (derivatives of 8-azapurin, pyrrolo[2,3-*d*]pyrimidine, pyrazolo[3,4-*d*]pyrimidine) and tricyclic (derivatives of benzo- guanine) [23,24]. The change regarding the necessity of the guanose system in the structure of guanosine analogues led to the synthesis of 3,9-dihydro-3-[(2-hydroxyethoxy)-methyl]-9-oxo-5*H*-imidazo[1,2-dihydroxy-1,2-*a*]purine derivatives that demonstrate antiviral activity (the so-called tricyclic acyclovir derivatives (TACV) or tricyclic ganciclovir derivatives (TGCV); Figure 1) [25,26,27] as well as the tricyclic modifications of compound RA-5021 and its enantiomer (A-5021), also involving the addition of an imidazole ring [28,29].

The mechanism of action of TACV and TGCV derivatives containing the imidazole ring has not been explained yet. It is known that thymidine kinase (TK) is necessary for their activation because TK^-^ strains do not show sensitivity to these substances. It has also been shown, in the example of some analogues, that they are effectively converted to triphosphates after exposure to pure HHV-1 TK and in a mixture also containing GMP (guanosine monophosphate) kinase and NDP (nucleoside diphosphate) kinase. It is clear, therefore, that thymidine kinase considers tricyclic analogues as a substrate for phosphorylation, although this process is less efficient than for parental acyclovir and ganciclovir. It is presumed that there is a lipophilic pocket in the thymidine kinase structure that allows the effective attachment of the derivatives of 6-phenyl TACV and TGCV [30].

Research on the relationship between the chemical structure and biological activity was performed for 20 C6-aryl-substituted TACV and TGCV analogues [31]. It was observed that the derivative activity depends on the structure and steric effects of the substituents at positions C6 and C7. The tolerance for substituent changes at C6 is very small. Only modifications to the substituent position on the *para* position of the phenyl moiety are allowed. The increase or preservation of TACV antiviral activity is generally observed after the methyl or methoxy group has been introduced into the TACV C6-phenyl ring para position. Both the introduction of these groups to the *ortho* or *meta* position as well as the introduction of a nitro group to the phenyl ring C6-TACV reduce the antiviral activity, with a simultaneous increase of the cytotoxic activity of [31,32,33,34,35,36,37]. The above studies allowed the selection 3,9-dihydro-3-[(2-hydroxyethoxy)methyl]-6-(4-methoxyphenyl)-9-oxo-5*H*-imidazo-[1,2-*a*]–purine (6-(4-MeOPh)-TACV) for the stability study as the most promising drug candidate. In order to improve the lipophilicity and bioavailability parameter of this group of compounds, the synthesis of their esters (acetyl, isobutyryl, pivaloyl, nicotinic, ethoxycarbonyl) as prodrugs was developed [38,39]. In acid stability studies, it was found that in both the 6-(4-MeOPh)-TACV esters and ACV esters, the steric factor plays a stabilizing role for the ester moiety. It has also been proven that the hydrolysis of ethoxycarbonyl esters occurs in a two-step reaction [40,41].

Thus, the aim of the presented research was to assess the susceptibility to enzymatic degradation (in vitro) of the tricyclic acyclovir derivative (6-(4-MeOPh)-TACV) and its esters (acetyl, isobutyryl, pivaloyl, nicotinic, ethoxycarbonyl) compared with the stability of analogue acyclovir esters (Figure 1). For this purpose, the studies were conducted in an environment of plasma proteins as factors catalyzing their hydrolytic degradation and in a plasma environment enriched with esterase from porcine liver (PLE). PLE is an enzyme imitating human esterase and allows provision of the same model of the prodrug metabolism reaction as in human plasma. The HPLC method with UV detection was used for the quantitative analysis of the reactions, and the HPLC-MS/MS method was used to identify the degradation products under the tested conditions.

## 2. Results and Discussion

Acetyl (Ac-), iso-butyryl (*i*But-), pivaloyl (Piv-), ethoxycarbonyl (Etc-), and nicotinoyl (Nic-) esters of acyclovir and their tricyclic derivatives were selected for the enzymatic stability studies. The tricyclic acyclovir derivative (3,9-Dihydro-3-[(2-hydroxyethoxy)methyl]-6-(4-methoxyphenyl)-9-oxo-5H-imidazo[1,2-*a*]–purine; 6-(4-MeOPh)-TACV) and its esters were obtained from the Poznan Institute of Bioorganic Chemistry Polish Academy of Sciences. The synthesis was carried out in laboratory conditions according to the literature [31,38]. In contrast, acyclovir esters were synthesized at the Chair and Department of Pharmaceutical Chemistry (Poznan University of Medical Sciences), also in a laboratory scale, according to the literature [40]. The previously published data also concern the determination of the lipophilicity parameter of the tested compounds using the HPLC technique and a comparison of the experimental values with the obtained computational techniques. The lipophilicity of the tested esters appeared as follows: Ac- < Nic- < Etc- < iBut- < Piv- [38,39].

### 2.1. HPLC Method Validation

Developed chromatographic separation conditions using the HPLC-RP technique (column: LiChrospher 100 RP-18, 250 × 4 mm, 10 μm) (Table 1) were used for the determination of all tested compounds (Figure 1) in plasma. These conditions guaranteed selective determination using an internal standard in the presence of plasma protein residues (Table 1).

The linearity study was performed for two dilution series in the concentration range 24–240 μg/mL (Supplementary Material, Table S1). The observed dependences of the size of the recorded signals as a function of the concentration of the tested compounds in the plasma were linear as described by the equation y = *b*x. The values of the correlation coefficients r ≥ 0.9972 indicated a strong correlation of the studied relationships. The calculated statistical parameters of the equations were used to determine the limits of detection (LOD: 5–16 μg/mL) and quantification (LOQ: 16–48 μg/mL) of the test compounds (Table S1).

To assess the precision, accuracy, and reproducibility of the assays by the developed method, the tested compounds in plasma for two solutions in significantly different concentrations of the analyte were marked (Tables S2 and S3). The tests were repeated the next day to estimate the repeatability of the results (between days and within the day). The values of the variation coefficients in the range of 1.17%–5.27% indicate the appropriate precision of the research methods (Tables S2 and S3). The coefficients of variation for repeatability were 0.38%–0.99%. The accuracy of the developed methods was assessed on the basis of recovery in the range of 89.8%–106.2% (Tables S2 and S3). The results of the statistical analyses confirm that the developed chromatographic conditions ensure precise, accurate, and reproducible analysis of the tested compounds in the plasma.

Analyte stability studies (supernatant) indicate that the smallest loss of the tested compound is observed during its storage in the freezer (−20 °C) and to a large extent depends on the type of substance and its concentration. Solutions with a lower concentration were proven to be more stable. Thus, the solutions of 6-(4-MeOPh)-TACV analyte and its esters, *i*But-, Etc-, and Nic-, and acyclovir esters, Etc-ACV and Piv-ACV, were considered stable under these conditions for 27 days of storage. In contrast, the analyte solutions of the remaining compounds were considered stable for 10 days of storage under freezing conditions. It is not recommended to store them at room temperature and in a refrigerator (4–8 °C) due to the observed gradual loss of the tested substance.

### 2.2. Enzymatic Stability

**Figure 2:**
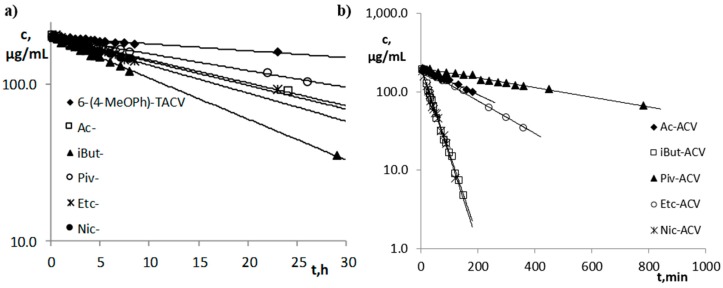
Semi-logarithmic plots of the *c_t_* = *f*(*t*) response of the hydrolysis reaction in plasma (80%, 37 °C): (**a**) 6-(4-MeOPh)-TACV and its esters; (**b**) acyclovir esters.

The condition for determining the values of the observed rate constants is to observe changes in the concentration of the tested compound over time ensuring degradation of at least 80% of the compound. In plasma, hydrolysis reactions were carried out until a minimum 50% loss of the tested compound was obtained except for the extremely stable 6-(4-MeOPh)-TACV under these conditions. In this case, the observed loss was 22% during the 25-h reaction. Further carrying out of this reaction in vitro in a temperature of 37 °C was not recommended (Figure 2). The *k_obs._* values were determined for the hydrolysis reaction of the tested compounds in human plasma (80%, 37 °C) from the linear dependence:

ln *C_t_* = ln *C*_0_ − *k_obs._·t*, (1)

demonstrating that the reaction is consistent in the first-order kinetics (towards the substrate concentration: *C*_0_, *C_t_*—at the beginning of the reaction and after time *t*, respectively) (Figure 2).

Studies on the susceptibility of compounds to hydrolysis in plasma have shown that the tricyclic derivatives of ACV (6(4-MeOPh)-TACV and its esters) are characterized by high stability under the above conditions (Figure 2a). The fastest decomposition occurred for the *i*But ester, for which t_0.5_ was 11.52 h (Table 2). The parallelism test showed that the difference in the stability of the Ac- and Etc- esters in the plasma is statistically insignificant. Susceptibility to hydrolysis in the plasma of tricyclic compounds can, therefore, be ranked as follows:

6(4-MeOPh)-TACV < Piv- < Ac- = Etc- < Nic- < *i*But-.

The *k_obs._* values for this group of compounds are in the range 2.81·10^−6^–1.67·10^−5^ s^−1^, while the t_0.5_ values in the range 11.52–68.40 h (Table 2).

The plasma stability of acyclovir esters (Figure 2b) is much lower than the 6(4-MeOPh)-TACV esters. The *k_obs._* values for this group of compounds are in the range 2.38·10^−5^–4.31·10^−4^ s^−1^, while the t_0.5_ values in the range 0.45–8.09 h (Table 2). The *k_obs._* values of ACV esters are 3.5–37-fold higher compared to the *k_obs._* values of the analogous esters of its tricyclic derivative. It was proven by the parallelism test that the observed difference in the susceptibility of *i*But-ACV and Nic-ACV esters to hydrolysis under the influence of plasma enzymes is statistically insignificant. Therefore, the ACV esters’ susceptibility to degradation in plasma can be ranked as follows:

Piv-ACV < Ac-ACV < Etc-ACV < *i*But-ACV = Nic-ACV.

**Scheme 1:**
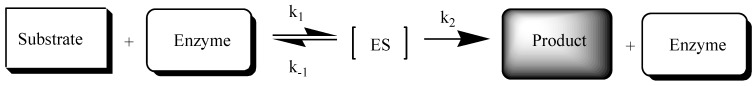
Scheme of the enzymatic reaction (ES—substrate complex with enzyme; *k*_1_, *k*_−1_, *k*_2_—partial reaction rate constants).

For the correct interpretation of the distribution of the tested compounds under the influence of esterase, it is necessary to know the specificity of the enzymatic reactions (Scheme 1). The biotransformation occurring during enzymatic hydrolysis can be treated as a limited capacity process and described using the Michaelis–Menten equation:

(2) $$\;V=-\frac{dc}{dt}=\frac{V_{m}\cdot c}{K_{M}+c},$$

where: *c*—substance concentration, *V* = −(*dc*/*dt*)—rate of change of substance concentration, *V_m_*—maximum theoretical speed of the process, and *K_M_*—Michaelis constant (numerically equal to the concentration of the substance at which its elimination occurs at a rate equal to half of *V_m_*).

In case the value of the Michaelis constant is much higher than the concentration of the substance (*K_M_* >> *c*), Equation (2) can be simplified to the form of:

(3) $$V=-\frac{dc}{dt}=\frac{k_{2}}{K_{M}}\cdot c_{E}\cdot c=\frac{V_{m}}{K_{M}}\cdot c=k_{obs.}\cdot c,$$

where: *c_E_*—enzyme concentration.

After transformation:

(4) $$k_{obs.}=\frac{k_{2}}{K_{M}}\cdot c_{E}=\frac{V_{m}}{K_{M}}.$$

The above situation takes place for processes occurring in accordance with the first order kinetics. The rate of change of the substance concentration is directly proportional to the theoretical maximum process speed and substance concentration. However, in the opposite situation when the concentration of the substance is much higher than the Michaelis constant (*c* >> *K_M_*), Equation (2) takes the form of:

(5) $$V=-\frac{dc}{dt}=V_{m}.$$

This equation applies to processes occurring in accordance with zero-order kinetics. For this situation, the rate of change in the concentration of the substance is constant, equal to the maximum speed, and does not depend on the concentration of the substance. It is noteworthy, however, that before the reaction reaches the maximum velocity *V_m_*, its increase occurs linearly in proportion to the increase in concentration, according to the first-order kinetics rules [42,43].

**Figure 3:**
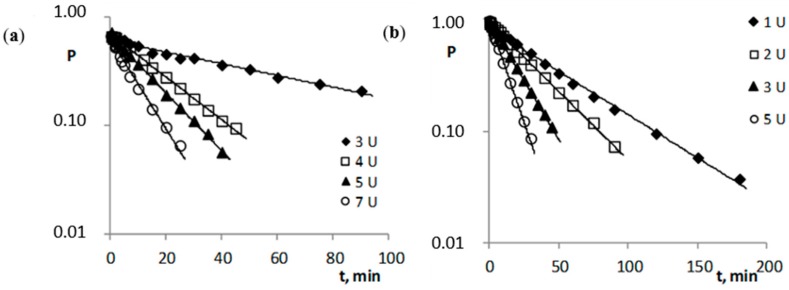
Semi-logarithmic plots of *P_t_* = *f*(*t*) reaction of the hydrolysis reaction of the pivaloyl esters of 6-(4-MeOPh)-TACV (**a**) and ACV (**b**) in plasma (80%, 37 °C) in the presence of porcine esterase.

The addition of porcine esterase significantly accelerated the decomposition, which explains the differences in the time reaction in Figure 2 and Figure 3. In this case, the esterase was used in an amount to provide an observation of the degradation of the tested compounds at a level not less than 50% for the lowest esterase concentration (Figure 3). In the presented studies, it was proven that, under the given reaction conditions, the distribution of all the tested compounds (c: tricyclic compounds 4.3·10^−4^–5.6·10^−4^ M; esters of ACV 6.0·10^−4^–7.4·10^−4^ M) in human plasma in the presence of esterase (c_E_ 2.75·10^−9^–6.05·10^−8^ M) occurs with first-order kinetics (Figure 3). Therefore, the rate of the enzymatic reaction can be described by Equations (3) and (4). The determined *k_obs._* values of the esterase-treated compounds (Table 3) are larger compared to the *k_obs._* values of the compounds only hydrolyzed in plasma (Table 2).

**Figure 4:**
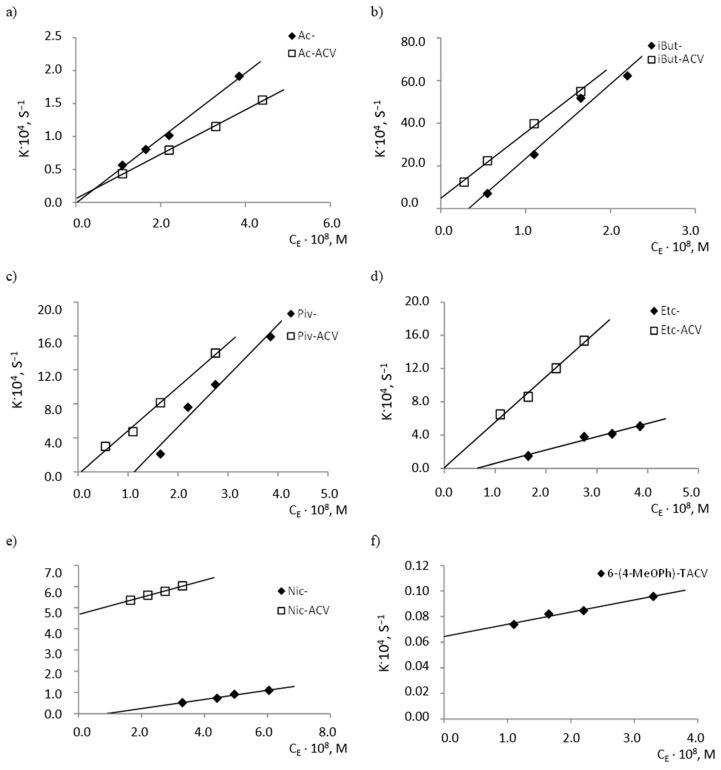
Plots of dependencies *k_obs._* = *f* (*c_E_*) of the hydrolysis reaction of (**a**) Ac- and Ac-ACV esters; (**b**) iBut- and iBut-ACV esters; (**c**) Piv and Piv-ACV esters; (**d**) Etc- and Etc-ACV esters; (**e**) Nic- and Nic-ACV esters; (**f**) 6-(4-MeOPh)-TACV compound in plasma (80%, 37 °C) in the presence of porcine esterase at 37 °C.

At the same time, with an increasing concentration of the enzyme, an increase in the rate of degradation reactions of the tested compounds was observed. It was also observed that the individual compounds differ in their susceptibility to hydrolysis by esterase (Figure 4), as indicated by the slope values of the analyzed lines *k_obs_.* = *f*(*c_E_*), which correspond to the quotient of the constant rate of *k*_2_ product creation and the speed of the Michaelis constant *K_M_* (*b* = *k*_2_/*K_M_*) (Table 4).

The least sensitive to hydrolysis under the influence of esterase was proven to be 6(4-MeOPh)-TACV, of which the *k*_2_/*K_M_* value (95.2 mol^−1^·L·s^−1^) was over 3000 times lower than for isobutyryl esters (Table 4). Because this compound has antiviral activity, its rapid degradation in the body would be a negative factor. The high resistance of the tricyclic acyclovir analogue to enzymatic hydrolysis suggests its slow metabolism and, therefore, the ability to obtain sufficiently high concentrations of substances in the blood for the required period of time.

It was further found that the difference in the degradation rate of the corresponding ACV esters and the esters of its tricyclic derivative in the presence of esterase is much smaller than in the case of the comparison of the hydrolysis rates of analogous pairs of esters in the plasma. Acyclovir esters in plasma decompose about 10 times faster than 6(4-MeOPh)-TACV esters, whereas in the presence of esterase, the ACV ester cleavage rate is only about 1.5 times higher than tricyclic derivatives (Table 3 and Table 4).

**Figure 5:**
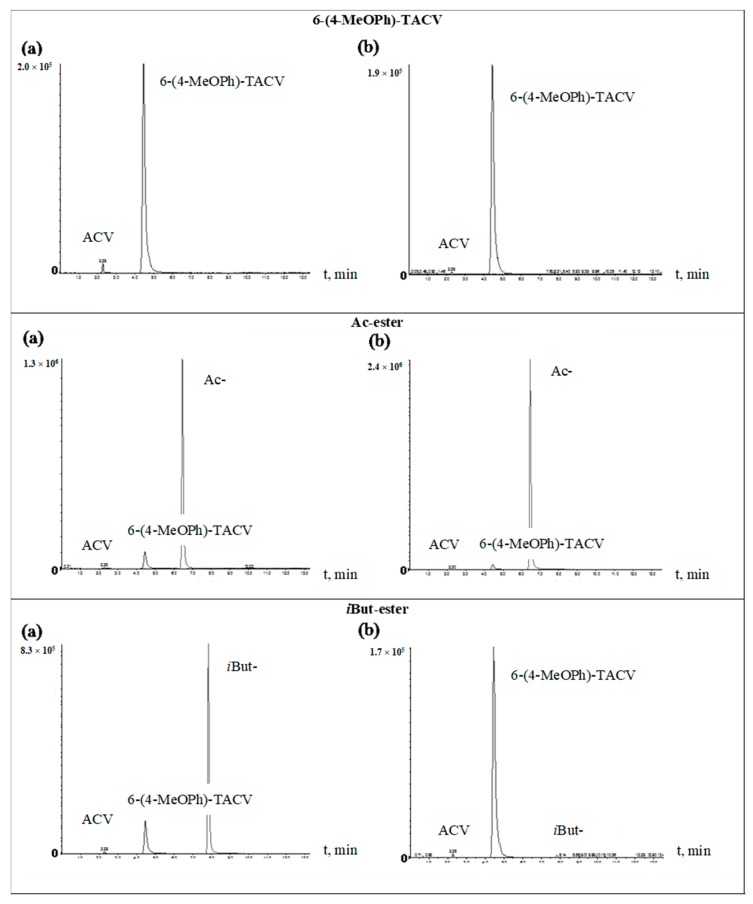
HPLC-MS/MS chromatograms of test compounds: 6-(4-MeOPh)-TACV and its Ac- and *i*But esters hydrolyzed at 37 °C. (**a**) in plasma (80%) for 24 h; (**b**) in plasma (80%) in the presence of porcine esterase (5.50·10^−9^ M) for 2 h.

**Figure 6:**
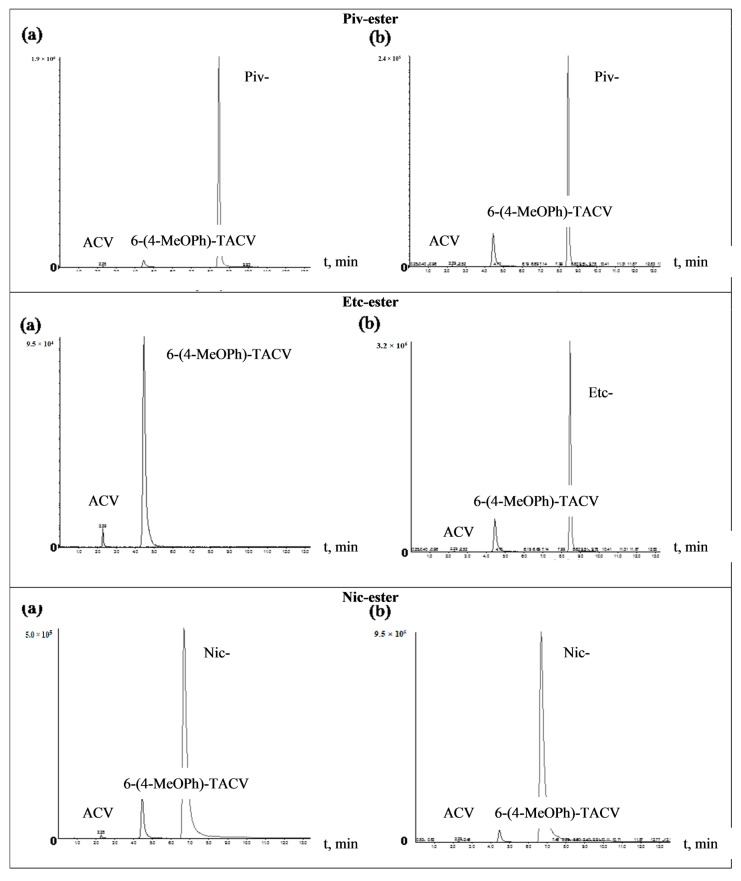
HPLC-MS/MS chromatograms of the 6-(4-MeOPh)-TACV (Piv-, Etc-, Nic-) ester hydrolyzed at 37 °C. (**a**) in plasma (80%) for 24 h; (**b**) in plasma (80%) in the presence of porcine esterase (5.50·10^−9^ M) for 2 h.

In order to identify 6(4-MeOPh)-TACV and ACV as tricyclic ester decomposition products in body fluids, an analysis was performed by the HPLC-MS/MS system. The tricyclic ACV analogue (6(4-MeOPh)-TACV) hydrolyzed in human plasma and in plasma with the addition of esterase was analyzed. The obtained results showed that, for all of the tested esters during their decomposition, 6(4-MeOPh)-TACV was formed (Figure 5 and Figure 6). Its amount was different depending on the esters’ decomposition rate under the given conditions and increased in proportion to the decrease of the ester signal. The degree of decomposition of individual esters and the formation of 6(4-MeOPh)-TACV, observed on HPLC-MS/MS chromatograms of the solutions hydrolyzed in plasma and in the presence of esterase, is close to the expected values calculated on the basis of the determined k_obs._ values. Due to the relatively low susceptibility of Ac- and Nic- esters to the decomposition by esterases, the solutions of these esters, treated with an enzyme of 5.50·10^−9^ M in 2 h, decomposed at an approximately two times lower rate than the analogous solutions incubated in plasma for 24 h (Figure 5 and Figure 6). Piv- ester, due to its greater sensitivity to the enzyme effect, was more strongly degraded by esterase than in the plasma itself (Figure 6). The most sensitive isobutyryl ester, on the other hand, was completely degraded in the presence of esterase as well as significant hydrolysis in the plasma (Figure 5). The hydrolysis of 6 (4-MeOPh)-TACV in both analyzed conditions was similar and occurred to a small extent, which can be explained by its high stability in plasma, and at the same time, its very low sensitivity to esterase activity (Figure 5; Table 3 and Table 4).

In the analyzed samples, the presence of small weakly visible signals of aciclovir were also observed even with the total distribution of the analyzed compound (*i*But-, Figure 5b). Their low intensity did not allow an approximate estimation of the amount of ACV formed. The obtained results prove that the main product of the decomposition of tricyclic ACV analogues in the plasma and in the presence of esterase is 6(4-MeOPh)-TACV. On the other hand, the ACV observed in the chromatograms in small amounts cannot be considered as a product of hydrolysis of the analyzed compounds (Figure 5 and Figure 6).

## 3. Experimental

### 3.1. Materials

3,9-Dihydro-3-[(2-hydroxyethoxy)methyl]-6-(4-methoxyphenyl)-9-oxo-5*H*-imidazo[1,2-*a*]–purine (6-(4-MeOPh)-TACV) and its esters, acetyl (Ac-), iso-butyryl (*i*But-), pivaloyl (Piv-), ethoxycarbonyl (Etc-), and nicotinoyl (Nic-), and esters of acyclovir (Ac-ACV, iBut-ACV, Piv-ACV, Etc-ACV, Nic-ACV) (Figure 1) were synthesized as previously reported at the laboratory scale [31,39,40]. Acyclovir (ACV) was received from the manufacturing company JELFA S.A. (Jelenia Góra, Poland). Acetonitrile for HPLC (isocratic basis) was from Avantor Performance Materials Poland S.A. (Gliwice, Poland) and nitrazepam (≥98.0%), sulfadimethoxin (≥98.5%), sulfafurazol (≥99.0%), and sulfathiazole (≥98.0%) from Sigma-Aldrich (Steinheim, Germany). Esterase from porcine liver (E2884 – 200 UN), for the enzymatic stability studies, was from Sigma-Aldrich (Steinheim, Germany). However, freshly frozen plasma (free of HIV, hepatitis, non-hemolyzed) was provided by the Regional Center for Blood Donation and Blood Treatment (Poznań, Poland).

### 3.2. Apparatus and Chromatographic Conditions

#### 3.2.1. HPLC-UV

Chromatographic analysis by HPLC-UV was performed using a Shimadzu LC-20AT system chromatograph (Kioto, Japan). As a stationary phase, a LiChrospher 100 RP-18 (250 × 4 mm, 10 μm; Merck, Darmstadt Germany) was used. To determine the content of the tested analogues in the plasma, a pre-column C18, 4 mm × 3.0 mm was used (Phenomenex, Aschaffenburg, Germany). The mobile phases were mixtures of acetonitrile-water with CH_3_COOH (20 mM) and KCl (1 mM) in different proportions (Table 1). The flow rate of the mobile phase was 1.0 mL/min, the injection volume was 20 µL, and the UV detection was carried out at 262 nm for the 6-(4-MeOPh)-TACV analogs and 254 nm for the ACV analogs. As internal standards, solutions of sulfadimethoxin, nitrazepam, sulfathiazole, or sulfafurazole in a mixture of acetonitrile-methanol (1:1, *v*/*v*) were used (Table 1). Separation was performed at 25 °C.

#### 3.2.2. HPLC-MS/MS

HPLC-MS/MS analyses of the 6-(4-MeOPh)-TACV and its esters were performed with the use of an Infinity 1260 HPLC chromatograph (Agilent Technologies, Santa Clara, CA, USA) interfaced to a 4000 QTRAP tandem mass spectrometer (AB Sciex, Framingham, MA, USA). The HPLC system was composed of a vacuum degasser, a binary pump, an autosampler, and a thermostated column oven. The mass spectrometer was equipped with an electrospray ionization (ESI) Turbo V^TM^ ion source.

Chromatographic separation was performed using a Lichrospher RP-18 (250 mm × 4 mm, 5 µm; Merck, Darmstadt, Germany) column and gradient elution. The mobile phase A consisted of water with addition of ammonium formate (5 mM) and the mobile phase B consisted of water with the addition of formic acid (0.1%, *v*/*v*) (Table 5). The flow rate and injection volume were set at 0.8 mL/min and 5 µL, respectively.

The separation temperature was maintained at 24 °C. The mass spectrometer was employed in positive ion mode, which led to pseudo-molecular ions of the analytes [M + H]^+^. The ion source parameters were the following: Ion spray voltage, 5500 V; source temperature, 550 °C; ion source gas 1, 50 psig; ion source gas 2, 60 psig; and curtain gas, 40 psig. A multiple reaction monitoring (MRM) scan type was applied, using nitrogen as the collision gas. Two transitions for each substance were selected. The MRM transitions and the related optimized parameters were the declustering potential (DP), collision energy (CE), and collision cell exit potential (CXP), and are shown in Table 6. Optimization of the parameters for the MS/MS analyses was performed by infusion of the standard solutions of 6-(4-MeOPh)-TACV, its esters, and ACV utilizing a syringe pump directly combined with the mass spectrometer. Data acquisition and processing were performed using Analyst 1.5 software (AB Sciex, Framingham, MA, USA).

#### 3.2.3. Other Equipment

In the tested compounds’ determination in plasma and their enzymatic stability studies, the micro centrifuge 200 (Hettich Zentrifugen, Germany) and the FBH 612 water bath (Fischerbrand, Schwere, Germany) were used.

Other equipment included an ultrasonic bath RK 510H (Bandelin Elektrolic, Berlin, Germany) and a filter for demineralized water USF 800 (Seradest, Berlin, Germany).

#### 3.2.4. Phosphate Buffer PBS pH 7.4

In total, 0.7738 g of sodium chloride, 0.0193 g of potassium chloride, 0.0250 g of potassium dihydrogen phosphate, and 0.1096 g of sodium hydrogenphosphate were weighed and dissolved in 95 mL of deionized water. The solution was thermostated at 37 °C for 24 h and adjusted to pH 7.4 with 1.5 mL of titrant solution (25 mM NaOH). After cooling to room temperature, the contents were quantitatively transferred to a volumetric flask and supplemented with deionized water to 100.0 mL.

### 3.3. HPLC Method Validation for Determination of the Tested Compounds in Plasma

#### 3.3.1. Stock Solutions

About 3.0 mg of the compound were weighed and dissolved in 0.50 mL of dimethyl sulfoxide (DMSO).

#### 3.3.2. Selectivity

In order to check the selectivity of the method for the implementation of the planned tests, solutions of the tested compounds in plasma (80%, *v*/*v*) with the addition of phosphate buffer solution pH 7.4 (PBS), thermostated for 2 h at 37 °C, were used, then mixed with appropriate internal standards (1:2, *v*/*v*) (Table 1). The mixture was centrifuged for 15 min (5 min, 13,000 rpm) and then the supernatants were applied to the column and chromatograms were recorded between 0 and 30 min.

#### 3.3.3. Linearity

A dilution series (Table 2) of the stock solutions of the tested compounds in plasma (80%, *v*/*v*) with the addition of PBS buffer was prepared. To 0.2 mL of the solution, 0.4 mL of the appropriate internal standard solution (Table 1) were added and mixed. The mixture was centrifuged for 15 min (5 min, 13,000 rpm) and then the supernatants were applied to the column and chromatograms were recorded. For each sample and series, the test was performed three times. Statistical parameters were determined according to the ratio of the areas of the peak of the tested compound peak area for the internal standard as a function of the concentration of the test substance. The values of the limit of detection (LOD) and determination (LOQ) were determined by using the formula:

(6) $$LOD\;\left(LOQ\right)=\frac{к\cdot SD_{a}}{b},$$

where *к* is 3.3 for LOD and 10 for LOQ, *SD_a_* is the standard deviation of the intercept, and *b* is the slope.

#### 3.3.4. Precision and Accuracy

The precision and accuracy of the method was assessed by determining the content of the test substances in plasma (80%, *v*/*v*) with the addition of PBS buffer at two different concentrations (Table 3 and Table 4). The study was performed in two days (inter-day, intra-day) and the test was repeated six times for each series of solutions.

#### 3.3.5. Stability of the Analyte

For each substance, samples with two concentrations in the linearity range of the method were prepared. The supernatants of both solutions were analyzed by HPLC immediately after preparation and after storage at room temperature (25 °C), in a refrigerator (4 °C) and freezer compartment (−20 °C). The degree of decomposition of the tested compounds with respect to the initial value was determined.

### 3.4. Enzymatic Stability of Tested Compounds

#### 3.4.1. Stability Test in Plasma

Stability tests were performed in human plasma (80%, *v/v*) thermostated in a water bath at 37 °C. In total, 2.50 mg of each of the analyzed compounds were weighed and dissolved in 0.5 mL of DMSO (5.0 mg/mL). For plastic screw-on tubes containing 4.0 mL of plasma, 0.8 mL of PBS buffer were added, mixed, and thermostated in a water bath. Then, 0.2 mL of the tested compound solution in DMSO (5.0 mg/mL) were added to the incubated plasma. Samples were thermostated at 37 °C and at specified intervals. After mixing, 0.2 mL of the test sample were taken and transferred to 0.4 mL of the appropriate internal standard solution (Table 1). After mixing, the samples were centrifuged for 15 min (5 min, 13,000 rpm). The supernatant was transferred to the tubes and analyzed by the HPLC method in a suitable phase system (Table 1). The initial concentration of the tested compounds in the plasma was 200 μg/mL, while in the analyte solution (supernatant) it was 66.7 μg/mL.

#### 3.4.2. Stability Test in Plasma with Esterase

A 1 U/μL aqueous pork esterase solution was prepared by diluting the suspension with 200 μL of water. Here, 168,000 Da was used as the molecular weight of the esterase and the molar concentration of the enzyme in a determined volume of the test sample of 5.0 mL was calculated (1 μL of the enzyme solution in 5.0 mL corresponded to a concentration of 5.50 × 10^−9^ M).

For plastic screw-on tubes containing 4.0 mL of plasma, 0.8 mL of PBS buffer was added, mixed, and thermostated in a water bath (37 °C). A fixed volume of enzyme (0.5–11.0 μL) was added to the incubated plasma, mixed, and thermostated for 15 min. Then, 0.2 mL of the tested compound solution in DMSO (5.00 mg/mL) were added, mixed thoroughly, and proceeded as described in Section 3.4.1.

#### 3.4.3. HPLC-MS/MS Analysis of Enzymatic Hydrolysis Products

The HPLC-MS/MS method was used for analysis of the solutions hydrolyzed in plasma (80% *v*/*v*, 37 °C, 24 h) and in plasma (80%, *v*/*v*) with porcine esterase at a concentration of 5.50 × 10^−9^ M (37 °C, 2 h). The samples were prepared according to the procedure described in Section 3.4.1 and Section 3.4.2, and then diluted with water in a proportion of 1:100 (*v*/*v*).

## 4. Conclusions

The obtained results lead to the conclusion that 6(4-MeOPh)-TACV esters are more susceptible to hepatic metabolism compared to ACV esters. By the HPLC-MS/MS method, it was confirmed that the main product of their hydrolysis in plasma is 6(4-MeOPh)-TACV and further degradation to ACV was not proven. Thus, the mechanism of action of tricyclic analogues does not result from their decomposition to ACV.

6(4-MeOPh)-TACV is a compound that is sufficiently stable under the above conditions tand thus able to achieve a satisfactory concentration in body fluids and not get metabolized too quickly. At the same time, it is noteworthy that the t_0.5_ values of the tested compounds in plasma (0.45–68.40 h) were found to be significantly lower than the t_0.5_ value used in the treatment of valaciclovir (226 h) [44]. Therefore, they can probably undergo transformation into the active substance in the body at a rate that makes it possible to obtain a therapeutic concentration.

## Figures and Tables

**Table 1:** Conditions for chromatographic separation of the HPLC-UV method and retention times of the tested compounds.

Compound	Mobile Phase	Flow Rate, mL/min	Retention Time, min	Internal Standard—i.s. μg/mL	Retention Time of i.s., min
	Acetonitrile—CH_3_COOH (2 mM), KCl (1 mM), *v*/*v*				
plasma residue 6-(4-MeOPh)-TACV Nic- Etc- *i*But-	35:65	1.0	2.01 4.29 8.16 10.83 13.49	sulfadimethoxin (192)	6.65
plasma residue 6-(4-MeOPh)-TACV Ac- Piv-			2.01 4.29 6.95 20.55	nitrazepam (192)	11.35
plasma residue ACV Ac-ACV	15:85	0.7	3.50 3.50 5.51	sulfathiazole (96)	9.72
plasma residue ACV *i*But-ACV Piv-ACV	23:77	1.0	1.59 2.47 5.02 7.89	sulfafurazol (112)	1.59
plasma residue ACV Etc-ACV	20:80	1.0	2.41 3.33 4.84	sulfafurazol (56)	2.41
plasma residue ACV Nic-ACV			2.41 3.33 4.16	sulfafurazol (112)	

**Table 2:** The observed pseudo-first-order rate constants for the hydrolysis of test compounds in plasma (80%, 37 °C).

Compound	k_obs._ ± Δk_obs._, s^−1^	t_0.5_, h	-r	n
6-(4-MeOPh)-TACV	(2.81 ± 0.36)·10^−6^	68.40	0.9822	13
Ac-	(9.36 ± 0.72)·10^−6^	20.57	0.9933	13
*i*But-	(1.67 ± 0.04)·10^−5^	11.52	0.9992	15
Piv-	(6.84 ± 0.33)·10^−6^	28.13	0.9977	12
Etc-	(9.74 ± 1.16)·10^−6^	19.77	0.9824	14
Nic-	(1.15 ± 0.13)·10^−5^	16.78	0.9837	14
Ac-ACV	(6.47 ± 0.49)·10^−5^	2.98	0.9912	16
*i*But-ACV	(4.16 ± 0.16)·10^−4^	0.46	0.9978	16
Piv-ACV	(2.38 ± 0.17)·10^−5^	8.09	0.9932	15
Etc-ACV	(7.99 ± 0.37)·10^−5^	2.41	0.9976	13
Nic-ACV	(4.31 ± 0.23)·10^−4^	0.45	0.9959	15

**Table 3:** The observed pseudo-first-order rate constants for hydrolysis of the tested compounds in plasma (80%, 37 °C) in the presence of porcine esterase.

Compound	c_E,_ U	c_E,_ M	k_obs._ ± Δk_obs._, s^−1^	t_0.5_	-r	n
6-(4-MeOPh)-TACV	2.0	1.10·10^−8^	(7.39 ± 0.49)·10^−6^	26.0 h	0.9950	13
	3.0	1.65·10^−8^	(8.21 ± 0.58)·10^−6^	23.5 h	0.9938	14
	4.0	2.20·10^−8^	(8.37 ± 0.24)·10^−6^	22.7 h	0.9988	16
	6.0	3.30·10^−8^	(9.58 ± 0.39)·10^−6^	20.1 h	0.9975	16
Ac-	2.0	1.10·10^−8^	(5.66 ± 0.36)·10^−5^	3.4 h	0.9940	16
	3.0	1.65·10^−8^	(8.04 ± 0.50)·10^−5^	2.4 h	0.9943	16
	4.0	2.20·10^−8^	(1.02 ± 0.05)·10^−4^	1.9 h	0.9966	16
	7.0	3.85·10^−8^	(1.91 ± 0.10)·10^−4^	1.0 h	0.9968	13
*i*But-	1.0	5.50·10^−9^	(7.05 ± 0.39)·10^−4^	16.4 min	0.9962	14
	2.0	1.10·10^−8^	(2.53 ± 0.14)·10^−3^	4.6 min	0.9978	10
	3.0	1.65·10^−8^	(5.17 ± 0.10)·10^−3^	2.2 min	0.9997	11
	4.0	2.20·10^−8^	(6.22 ± 0.45)·10^−3^	1.9 min	0.9962	10
Piv-	3.0	1.65·10^−8^	(2.08 ± 0.19)·10^−4^	55.5 min	0.9908	13
	4.0	2.20·10^−8^	(7.59 ± 0.18)·10^−4^	15.2 min	0.9993	14
	5.0	2.75·10^−8^	(1.03 ± 0.03)·10^−3^	11.2 min	0.9992	13
	7.0	3.85·10^−8^	(1.59 ± 0.11)·10^−3^	7.3 min	0.9936	14
Etc-	3.0	1.65·10^−8^	(1.48 ± 0.07)·10^−4^	1.3 h	0.9972	13
	5.0	2.75·10^−8^	(3.79 ± 0.27)·10^−4^	30.5 min	0.9930	15
	6.0	3.30·10^−8^	(4.15 ± 0.26)·10^−4^	27.8 min	0.9964	11
	7.0	3.85·10^−8^	(5.07 ± 0.43)·10^−4^	22.8 min	0.9927	12
Nic-	6.0	3.30·10^−8^	(5.24 ± 0.38)·10^−5^	3.7 h	0.9933	14
	8.0	4.40·10^−8^	(7.31 ± 0.49)·10^−5^	2.6 h	0.9943	14
	9.0	4.95·10^−8^	(9.18 ± 0.65)·10^−5^	2.1 h	0.9937	14
	11.0	6.05·10^−8^	(1.10 ± 0.17)·10^−4^	1.7 h	0.9764	12
Ac-ACV	2.0	1.10·10^−8^	(4.37 ± 0.27)·10^−5^	4.4 h	0.9946	15
	4.0	2.20·10^−8^	(7.94 ± 0.23)·10^−5^	2.4 h	0.9988	15
	6.0	3.30·10^−8^	(1.15 ± 0.02)·10^−4^	1.7 h	0.9995	15
	8.0	4.40·10^−8^	(1.55 ± 0.04)·10^−4^	1.2 h	0.9992	15
*i*But-ACV	0.5	2.75·10^−9^	(1.24 ± 0.12)·10^−3^	9.3 min	0.9869	15
	1.0	5.50·10^−9^	(2.24 ± 0.09)·10^−3^	5.2 min	0.9971	17
	2.0	1.10·10^−8^	(3.98 ± 0.13)·10^−3^	2.9 min	0.9985	16
	3.0	1.65·10^−8^	(5.49 ± 0.20)·10^−3^	2.1 min	0.9979	16
Piv-ACV	1.0	5.50·10^−9^	(2.97 ± 0.09)·10^−4^	38.9 min	0.9988	16
	2.0	1.10·10^−8^	(4.71 ± 0.10)·10^−4^	24.5 min	0.9995	16
	3.0	1.65·10^−8^	(8.11 ± 0.15)·10^−4^	14.3 min	0.9995	15
	5.0	2.75·10^−8^	(1.40 ± 0.04)·10^−3^	8.3 min	0.9989	15
Etc-ACV	2.0	1.10·10^−8^	(6.48 ± 0.35)·10^−4^	17.8 min	0.9960	15
	3.0	1.65·10^−8^	(8.60 ± 0.34)·10^−4^	13.4 min	0.9977	16
	4.0	2.20·10^−8^	(1.20 ± 0.03)·10^−3^	9.6 min	0.9991	15
	5.0	2.75·10^−8^	(1.54 ± 0.07)·10^−3^	7.5 min	0.9971	15
Nic-ACV	3.0	1.65·10^−8^	(5.35 ± 0.23)·10^−4^	21.6 min	0.9971	16
	4.0	2.20·10^−8^	(5.59 ± 0.50)·10^−4^	20.7 min	0.9892	15
	5.0	2.75·10^−8^	(5.77 ± 0.32)·10^−4^	20.0 min	0.9966	13
	6.0	3.30·10^−8^	(6.03 ± 0.22)·10^−4^	19.2 min	0.9981	15

**Table 4:** Parameters describing the dependence of *k_obs._* = *f*(*c_E_*) of the hydrolysis reaction of the tested compounds in human plasma in the presence of the porcine esterase (37 °C).

Compound	*k*_2_/*K_M_*, mol ^−1^·L·s^−1^	Compound	*k*_2_/*K_M_*, mol ^−1^·L·s^−1^
6-(4-MeOPh)-TACV	(9.52 ± 4.26)·10^1^		
Ac-	(4.89 ± 0.64)·10^3^	Ac-ACV	(3.54 ± 0.18)·10^3^
*i*But-	(2.80 ± 1.00)·10^5^	*i*But-ACV	(3.48 ± 0.30)·10^5^
Piv-	(3.66 ± 1.93)·10^4^	Piv-ACV	(4.97 ± 0.89)·10^4^
Etc-	(1.28 ± 0.51)·10^4^	Etc-ACV	(5.52 ± 1.02)·10^4^
Nic-	(1.77 ± 0.47)·10^3^	Nic-ACV	(4.04 ± 0.79)·10^3^

**Table 5:** The composition of the mobile phase in the time intervals of the gradient elution used in the HPLC-MS/MS method.

Time [min]	Phase A [%]	Phase B [%]
0.0	65.0	35.0
1.0	65.0	35.0
8.0	15.0	85.0
10.0	15.0	85.0
11.0	65.0	35.0
17.0	65.0	35.0

**Table 6:** Parameters optimized for the MS/MS analyses.

Compound	Molecular Weight, Da	Retention Time, min	Precursor Ion, *m*/*z*	Product Ions, *m*/*z*	DP, V	CE, V	CXP, V	DT, ms
ACV	225.2	2.29	226.2	152.1	51	20	13	57
				110.1		43	10	
6-(4-MeOPh)-TACV	355.35	4.46	356.3	294.2	50	24	16	
				282.1		29	15	
Ac-	397.39	6.46	398.2	294.3	85	27	17	
				87.1		46	15	
*i*But-	425.45	7.82	426.2	294.3	100	28	17	
				115.2		42	20	
Piv-	439.47	8.45	440.5	294.3	112	27	24	
				129.3		45	22	
Etc-	427.42	7.36	428.2	194.3	110	27	17	
				117.3		31	21	
Nic-	460.45	6.68	461.2	294.3	100	30	17	
				150.2		41	27	

DP—declustering potential; CE—collision energy; CXP—collision cell exit potential; DT—dwell time.

## Supplementary Materials

The following are available online: **Table S1.** LOD, LOQ and ranges of HPLC methods for the determination of test compounds; **Table S2.** Precision and accuracy of the determinations of 6-(4-MeOPh)-TACV esters (*n* = 6); **Table S3.** Precision and accuracy of the determinations of acyclovir esters (*n* = 6).
