# Supplementary material for: Tricyclic Derivative of Acyclovir and Its Esters in Relation to the Esters of Acyclovir Enzymatic Stability: Enzymatic Stability Study
Source: Molecules. 2020 May 5;25(9):2156. doi: 10.3390/molecules25092156 (PMC7249156; doi:10.3390/molecules25092156)
Supplement: Supplementary file 1 [file molecules-25-02156-s001.pdf]

Tricyclic Derivative of Acyclovir and Its Esters in Relation to the Esters of Acyclovir Enzymatic Stability – Enzymatic Stability Study

Izabela Muszalska-Kolos [1,*], Monika A. Lesniewska-Kowiel [1], Szymon Plewa [2] and Agnieszka Klupczyńska [2]

[1] Chair and Department of Pharmaceutical Chemistry, Poznan University of Medical Sciences, 6 Grunwaldzka Str., 60-780 Poznań, Poland
[2] Chair and Department of Inorganic and Analytical Chemistry, Poznan University of Medical Sciences, 6 Grunwaldzka Str., 60-780 Poznań, Poland
* Correspondence: imuszals@ump.edu.pl; Tel. +48-61-854-6615; Fax: +48-61-854-6652

Validation parameters of the HPLC-UV method for determination of test compounds

HPLC-UV

Shimadzu LC-20AT system chromatograph (Kioto, Japan).
Stationary phase: a LiChrospher 100 RP-18 (250 × 4 mm, 10 µm; Merck, Darmstadt Germany
pre-column C18, 4 mm × 3.0 mm (Phenomenex, Aschaffenburg, Germany).
The mobile phases were the mixtures of acetonitrile-water with CH_3COOH (20 mM) and KCl (1 mM) in different proportions
Temp. – 25°C.
The flow rate of the mobile phase - 1.0 mL/min
Injection - 20 µL
UV detection - 262 nm for 6-(4-MeOPh)-TACV analogs and 254 nm for ACV analogs.
Internal standards, solutions of sulfadimethoxin, nitrazepam, sulfathiazole or sulfafurazole in a mixture of acetonitrile-methanol (1:1, *v/v*).

**Compound**	**Mobile Phase**<br>**Acetonitrile – CH_3COOH (2 mM), KCl (1 mM), v/v**	**Flow Rate, mL/min**
Nic-<br>Etc-<br>*i*But-	35:65	1.0
Ac-<br>Piv-		
Ac-ACV	15:85	0.7
*i*But-ACV<br>Piv-ACV	23:77	1.0
Etc-ACV<br>Nic-ACV	20:80	1.0

Table S1. LOD, LOQ and ranges of HPLC methods for the determination of test compounds.

Compound	Series	LOD, μg/mL	LOQ, μg/mL	y = *b*x	r	Range, μg/mL	Range, M
6-(4-MeOPh)-TACV	I	16	48	y = (3.99 ±0.36) ·10^{-3} ·x	0,9979	48 – 240	(1.33 – 6.67) ·10^{-4}
	II	9	27	y = (3.70 ±0.16) ·10^{-3} ·x	0,9991	48 – 240	(1.33 – 6.67) ·10^{-4}
Ac-	I	10	30	y = (4.44 ±0.21) ·10^{-3} ·x	0,9989	48 – 240	(1.22 – 6.04) ·10^{-4}
	II	5	16	y = (4.47 ±0.12) ·10^{-3} ·x	0,9996	24 – 240	(0.61 – 6.04) ·10^{-4}
*i*But-I	I	14	42	y = (3.78 ±0.27) ·10^{-3} ·x	0,9979	48 – 240	(1.13 – 5.64) ·10^{-4}
	II	16	48	y = (3.50 ±0.26) ·10^{-3} ·x	0,9972	48 – 240	(1.13 – 5.64) ·10^{-4}
Piv-I	I	8	23	y = (3.54 ±0.02) ·10^{-3} ·x	0,9991	24 – 240	(0.55 – 5.46) ·10^{-4}
	II	16	48	y = (3.99 ±0.32) ·10^{-3} ·x	0,9973	48 – 240	(1.09 – 5.46) ·10^{-4}
Etc-	I	8	24	y = (3.30 ±0.15) ·10^{-3} ·x	0,9992	24 – 240	(0.56 – 5.62) ·10^{-4}
	II	15	44	y = (3.40 ±0.22) ·10^{-3} ·x	0,9977	48 – 240	(1.12 – 5.62) ·10^{-4}
Nic-	I	12	36	y = (4.75 ±0.26) ·10^{-3} ·x	0,9988	48 – 240	(1.04 – 5.21) ·10^{-4}
	II	13	40	y = (5.07 ±0.31) ·10^{-3} ·x	0,9980	48 – 240	(1.04 – 5.21) ·10^{-4}
Ac-ACV	I	8	23	y = (4.09 ±0.16) ·10^{-3} ·x	0,9991	24 – 240	(0.89 – 8.92) ·10^{-4}
	II	8	23	y = (4.10 ±0.15) ·10^{-3} ·x	0,9991	24 – 240	(0.89 – 8.92) ·10^{-4}
*i*But-ACV	I	7	22	y = (3.66 ±0.17) ·10^{-3} ·x	0,9991	24 – 192	(0.81 – 6.46) ·10^{-4}
	II	6	17	y = (3.72 ±0.11) ·10^{-3} ·x	0,9995	24 – 240	(0.81 – 8.08) ·10^{-4}
Piv-ACV	I	12	36	y = (4.30 ±0.24) ·10^{-3} ·x	0,9984	48 – 240	(1.54 – 7.71) ·10^{-4}
	II	14	43	y = (4.84 ±0.31) ·10^{-3} ·x	0,9978	48 – 240	(1.54 – 7.71) ·10^{-4}
Etc-ACV	I	14	41	y = (1.47 ±0.09) ·10^{-3} ·x	0,9980	48 – 240	(1.60 – 8.02) ·10^{-4}
	II	8	24	y = (1.48 ±0.06) ·10^{-3} ·x	0,9991	24 – 240	(0.80 – 8.02) ·10^{-4}
Nic-ACV	I	6	17	y = (5.06 ±0.14) ·10^{-3} ·x	0,9995	24 – 240	(0.72 – 7.23) ·10^{-4}
	II	7	22	y = (4.65 ±0.16) ·10^{-3} ·x	0,9992	24 – 240	(0.72 – 7.23) ·10^{-4}

Table S2. Precision and accuracy of the determinations of 6-(4-MeOPh)-TACV esters (n = 6).

Compound	c, μg/mL	Day	$c_{det.}$ μg/mL	Accuracy, %	RSD, %
6-(4-MeOPh)-TACV	96.0	1	96.6 ±2.8	100.6 ±3.0	2.81%
		2	95.9 ±3.7	99.9 ±3.9	3.89%
	192.0	1	190.5 ±2.3	99.2 ±1.2	1.17%
		2	193.8 ±4.4	100.9 ±2.3	2.28%
Ac-	72.0	1	72.8 ±1.4	101.2±2.0	1.86%
		2	72.2 ±0.9	100.3 ±1.3	1.26%
	192.0	1	190.0 ±5.2	99.0 ±2.7	2.63%
		2	193.8 ±2.5	100.9 ±1.3	1.31%
*i*But-	96.0	1	101.2 ±2.5	105.5 ±2.6	2.36%
		2	101.2 ±3.3	105.5 ±3.4	3.36%
	192.0	1	194.7 ±3.1	101.4 ±1.6	1.50%
		2	194.0 ±4.8	101.0 ±2.5	2.50%
Piv-	96.0	1	96.0 ±2.3	100.0 ±2.4	2.32%
		2	97.1 ±2.9	101.2 ±3.0	2.99%
	120.0	1	107.8 ±3.4	89.8 ±2.8	2.98%
		2	118.7 ±3.9	98.9 ±3.2	3.28%
Etc-	72.0	1	73.6 ±1.1	102.3 ±1.6	1.48%
		2	74.6 ±2.9	103.6 ±4.0	3.86%
	120.0	1	115.4 ±3.6	96.2 ±3.0	2.98%
		2	121.1 ±1.3	100.9 ±1.1	1.05%
Nic-	72.0	1	73.9 ±1.9	102.7 ±2.7	2.50%
		2	73.6 ±1.8	102.3 ±2.4	2.39%
	120.0	1	116.6 ±3.5	97.1 ±2.9	2.85%
		2	124.2 ±4.0	103.5 ±3.4	3.26%

$c_{det.}$ - substance concentration determined by HPLC; RSD - coefficient of variation

Table S3. Precision and accuracy of the determinations of acyclovir esters (n = 6).

Compound	c, μg/mL	Day	$c_{det.}$ μg/mL	Accuracy, %	RSD, %
Ac-ACV	48.0	1	46.4 ± 1.9	96.8 ± 4.0	3.97%
		2	49.5 ± 1.6	103.0 ± 3.3	3.24%
	192.0	1	184.7 ± 4.3	96.2 ± 2.2	2.20%
		2	186.9 ± 4.1	97.3 ± 2.2	2.21%
*i*But-ACV	72.0	1	66.6 ± 1.3	92.5 ± 1.9	1.92%
		2	70.7 ± 1.7	98.1 ± 2.4	2.43%
	144.0	1	142.1 ± 4.0	98.7 ± 2.8	2.67%
		2	143.2 ± 2.2	99.5 ± 1.5	1.53%
Piv-ACV	48.0	1	46.6 ± 2.2	97.0 ± 4.6	4.48%
		2	47.7 ± 1.8	99.4 ± 3.8	3.82%
	120.0	1	115.3 ± 2.5	96.1 ± 2.1	2.08%
		2	118.1 ± 1.7	98.4 ± 1.4	1.46%
Etc-ACV	48.0	1	49.8 ± 2.8	103.8 ± 5.7	5.27%
		2	48.7 ± 2.7	101.5 ± 5.6	5.51%
	144.0	1	152.9 ± 1.5	106.2 ± 1.0	0.91%
		2	146.9 ± 3.7	102.0 ± 2.5	2.50%
Nic-ACV	72.0	1	68.7 ± 1.9	95.5 ± 2.6	2.59%
		2	72.4 ± 1.9	100.6 ± 2.7	2.64%
	144.0	1	144.8 ± 2.4	100.6 ± 1.7	1.61%
		2	145.6 ± 2.1	101.1 ± 1.5	1.46%

$c_{det.}$ - substance concentration determined by HPLC; RSD - coefficient of variation
